# Resident loneliness, social isolation and unplanned emergency department visits from supportive living facilities: a population-based study in Alberta, Canada

**DOI:** 10.1186/s12877-021-02718-5

**Published:** 2022-01-03

**Authors:** Stephanie A. Chamberlain, Susan E. Bronskill, Zoe Hsu, Erik Youngson, Andrea Gruneir

**Affiliations:** 1 Department of Family Medicine, Faculty of Medicine & Dentistry, University of Alberta, 6-50 University Terrace, Edmonton, Alberta T6G 2T4 Canada; 2 ICES, Toronto, Ontario Canada; 3 Management & Evaluation, Dalla Lana School of Public Health, Institute of Health Policy, University of Toronto, Toronto, Ontario Canada; 4 Sunnybrook Research Institute, Sunnybrook Health Sciences Centre, Toronto, Ontario Canada; 5 Women’s College Research Institute, Women’s College Hospital, Toronto, Ontario Canada; 6 Data and Research Services, Alberta SPOR Support Unit and Provincial Research Data Services, Alberta Health Services, Edmonton, Alberta Canada

**Keywords:** Supportive living, Emergency department visits, Loneliness, Social isolation

## Abstract

**Background:**

Supportive living (SL) facilities are intended to provide a residential care setting in a less restrictive and more cost-effective way than nursing homes (NH). SL residents with poor social relationships may be at risk for increased health service use. We describe the demographic and health service use patterns of lonely and socially isolated SL residents and to quantify associations between loneliness and social isolation on unplanned emergency department (ED) visits.

**Methods:**

We conducted a retrospective cohort study using population-based linked health administrative data from Alberta, Canada. All SL residents aged 18 to 105 years who had at least one Resident Assessment Instrument-Home Care (RAI-HC) assessment between April 1, 2013 and March 31, 2018 were observed. Loneliness and social isolation were measured as a resident indicating that he/she feels lonely and if the resident had neither a primary nor secondary caregiver, respectively. Health service use in the 1 year following assessment included unplanned ED visits, hospital admissions, admission to higher levels of SL, admission to NH and death. Multivariable Cox proportional hazard models examined the association between loneliness and social isolation on the time to first unplanned ED visit.

**Results:**

We identified 18,191 individuals living in Alberta SL facilities. The prevalence of loneliness was 18% (*n* = 3238), social isolation was 4% (*n* = 713). Lonely residents had the greatest overall health service use. Risk of unplanned ED visit increased with loneliness (aHR = 1.10, 95% CI: 1.04–1.15) but did not increase with social isolation (aHR = 0.95, 95% CI: 0.84–1.06).

**Conclusions:**

Lonely residents had a different demographic profile (older, female, cognitively impaired) from socially isolated residents and were more likely to experience an unplanned ED visit. Our findings suggest the need to develop interventions to assist SL care providers with how to identify and address social factors to reduce risk of unplanned ED visits.

**Supplementary Information:**

The online version contains supplementary material available at 10.1186/s12877-021-02718-5.

## Background

Many jurisdictions have seen a growth in assisted or supportive living (SL), settings designed to bridge the gap between independent living and institutional nursing home (NH) care [1]. Similar to assisted living in the United States, SL in Canada varies in terms of provincial standards and regulations, staffing, service availability, and discharge criteria [2]. In several Canadian provinces, SL is a part of the publicly funded continuing care system, with some (but not all) costs covered through provincial health insurance. What distinguishes SL from private retirement homes is that entry to SL is based on provincially determined assessment criteria for unmet needs and requires routine monitoring of resident quality of care [3]. Both US and Canadian research has shown that SL residents are similar to NH residents in terms of frailty, functional limitations, chronic conditions, antipsychotic use, and cognitive impairment; however, the setting is not designed to provide the services that are available in NHs [1, 4–7].

Few studies have examined the social well-being of SL residents [8]. SL is a unique environment in which to understand the intersections between social connection and health service use because it is situated in a liminal space between home and institutional care setting. Residents are in unfamiliar surroundings and routines, they must maintain some independence yet they must relinquish some autonomy [9]. Both loneliness and social isolation are of particular concern in SL settings, which typically function on the assumption that family members or friends are available to provide emotional and task-based support (e.g., shopping, transportation, financial management, advocacy) [10–14]. As well, SL facilities tend to offer fewer recreation and social activities than NHs potentially increasing the likelihood of loneliness and further isolation [15].

Research on loneliness, involvement in social activities, and quality of social relationships among SL residents is important because is it associated with health service use including increased risk of NH placement and hospitalization [6]. A systematic review found strong evidence for the association between social isolation and more frequent hospital readmissions and increased length of hospital stay [16]. Individual studies found that increased loneliness was associated with more frequent emergency department (ED) use compared to those who were not lonely [17, 18]. These studies suggest that loneliness and social isolation are associated with greater use of health services. At the same time, other research suggests that loneliness and social isolation may lead to the under-use of health services because of access challenges (e.g., available family or friends to assist with transportation or provide additional financial resources). This in turn, increases the likelihood of unmet needs [19]. These opposing findings point to an important gap in our understanding of the influence of loneliness, social isolation, and health service use more generally. Although the ED represents only one setting in the spectrum of care for older adults, ED visits can have serious negative health implications including pressure ulcers, delirium, and increased likelihood of developing new or worsening disability [20, 21]. Older adults (> 65 years) use EDs more than any other age group, however much of the published work on older adults use of ED does not distinguish those living in SL settings [22]. Understanding the occurrence of ED visits among individuals from SL, and identifying social factors that might contribute to those ED visits, can improve resident quality of care and the ways that health systems are used.

In this study, we used linked population-based health administrative and resident assessment data to describe the demographic and health service use patterns between lonely and socially isolated residents and to quantify associations between loneliness and social isolation on unplanned ED visits, among SL residents in Alberta, Canada.

## Methods

This retrospective cohort study of SL residents used population-based linked health administrative data from Alberta, Canada. The Resident Assessment Instrument-Home Care (RAI-HC) is administered to all publicly funded SL residents and is completed at resident admission and annually thereafter [3]. The RAI-HC is completed by a facility case manager and collects data on resident demographics, clinical characteristics, and functional status. Data on ED use comes from the National Ambulatory Care Reporting System (NACRS) and hospitalizations from the Discharge Abstract Database (DAD). The Alberta Health Care Insurance Plan (AHCIP) Provincial Registry was used to ensure residents were eligible for Alberta health care coverage and to capture all-cause mortality. All project data were accessed from the Alberta Health Services Enterprise Data Warehouse with support provided by the Alberta SPOR Support Unit Data and Research Services team (https://absporu.ca). Ethics approval was granted from the University of Alberta Research Ethics Board (Reference #: Pro00092070). The need for written and verbal consent from participants was waived by the University of Alberta Research Ethics Board because the study used existing electronic clinical health records (i.e., secondary analysis of health administrative data). These records (RAI-HC, DAD, NACRS) are used in Canada and elsewhere for public reporting, quality measurement, and research.

### Study cohort

We identified all SL residents (*N* = 18,191) who had at least one RAI-HC assessment between April 1, 2013 and March 31, 2018. We examined all SL residents who were between 18 and 105 years of age. We excluded a total of 1762 individuals, including those who did not have AHCIP coverage in the year of index assessment (*n* = 1754), and 8 who were missing age, sex, and/or valid health card number. For each resident, we used the first available assessment (index assessment) in the observation period for our analysis.

### Loneliness and social isolation

Loneliness reflects a discrepancy between desired and actual social connections and it is the emotional reaction to insufficient social relationships [23–25]. Using variables in the RAI-HC, we defined loneliness as a resident indicating that he/she feels lonely (F3b = 1) [26]. Social isolation is less well defined and does not have agreed upon measurement, but commonly assesses the size of a person’s social network [27, 28]. It reflects a limited reserve of support to draw on when in need [29]. The RAI-HC contains one variable that assesses the presence of a caregiver (does the resident have a primary or secondary caregiver). This caregiver refers to a family or friend caregiver (informal, unpaid) outside the facility. For the purposes of this study, we defined residents as socially isolated if they had neither a primary nor secondary caregiver (G1e a/b = 2). This variable was selected to measure social isolation because the lack of either a primary or secondary caregiver reflects the size of their immediate care network [27]. The limited availability of a close informal caregiver is consistent with definitions of social isolation and is one of the only measures of resident social network and support in the RAI-HC. We created a three-category variable (lonely, socially isolated, and neither lonely nor socially isolated) to characterize residents. Less than 1% (*n* = 101) of the cohort were both lonely and socially isolated. Rather than remove these individuals due to small cell sizes we elected to group them with the socially isolated group. This decision was made by first examining the descriptive and modelling results if this group were included with lonely only and then with socially isolated only. There were no differences with either approach, therefore we further examined the demographic characteristics of the both lonely and socially isolated group, comparing factors such as age, sex, functional status, and disease diagnoses. We found that those who were both lonely and socially isolated were comparable to the socially isolated only group rather than the lonely only group so we elected to group them with this socially isolated group.

### Health service use

We looked at health service use in the 1 year following the RAI-HC assessment and looked at the number of unplanned ED visits, hospital admissions, total number of days in hospital, total number of days in alternate level of care (ALC), admission to higher level of SL, admission to NH and all-cause mortality.

### Resident characteristics

We identified the level of support provided to individuals within each SL type. Publicly funded SL in Alberta, known as designated SL, is composed of three progressive levels of support moving from the lowest level of support available (SL3) to higher levels of support (SL4, SL4-Dementia) [30]. SL3 is for individuals who are medically and physically stable and can move independently or move with limited assistance. Health care aides are available on site 24 h a day and other health care services are scheduled and provided by home care. SL4 is for individuals with more complex health needs and who might require assistance with eating and transfers. SL4 has health care aides and a licensed practical nurse available 24 h a day, and other care needs (e.g., rehabilitation therapy) are contracted through home care. SL4D is like SL4 but is specifically for individuals with moderate to severe dementia.

Resident demographics from the RAI-HC included age (continuous), sex (female, male), marital status (single, married, widowed, separated or divorced). We included scales derived from the RAI-HC data including, the Cognitive Performance Scale [31], the Depression Rating Scale [32], Activities of Daily living [33], Instrumental Activities of Daily Living [34], and the Changes in Health, End-stage disease and Symptoms and Signs scale (CHESS) [35]. Higher scores in each scale indicates worsening performance or health instability. Other variables from the RAI-HC included a diagnosis of Alzheimer’s or other dementia, any psychiatric diagnosis (yes/no), number of medications, and number of falls.

### Analysis

We calculated descriptive statistics for resident demographic characteristics, functional status, disease diagnoses, and subsequent health system use. Multivariable Cox proportional hazard models examined the association between loneliness and social isolation on time to first unplanned ED visit, with a competing risks regression from Fine and Gray’s proportional sub-hazards model [36]. In the models, death, admission to a higher level of SL, or admission to NH were treated as competing risks. Residents were censored if at the end of the 1 year observation period they experienced no event (ED visit), remained in the same level of SL, or did not die. All analysis was conducted in SAS 9.4 (SAS Institute, Cary, NC). We received research ethics approval from the University of Alberta Research Ethics Board (Reference #: Pro00092070). Data used and analyzed during the current study are stored in the Alberta Health Services Enterprise Data Warehouse with support provided by the Alberta SPOR Support Unit Data and Research Services team in accordance with the provincial Health System Access and Data Cooperation Agreement between University of Alberta researchers and Alberta Health Services. All the methods were carried out in accordance with relevant guidelines and regulations stipulated by the University of Alberta Research Ethics Board and the Alberta Health Services Data Cooperation Agreement.

## Results

We identified 18,191 individuals living in Alberta SL facilities from April 1, 2013 to March 31, 2018. The majority were identified in SL4 (*n* = 10,693, 58.8%) followed by SL4D (*n* = 4703, 25.9%) and SL3 (*n* = 2795, 15.4%). We found that 18% of residents were lonely (*n* = 3238), 4 % were socially isolated (*n* = 713), and 78% were neither lonely nor socially isolated (*n* = 14,240). The prevalence of loneliness and social isolation decreased as the level of SL support increased (Table 1).

**Table 1:** Characteristics, functional status, and disease diagnoses of SL residents in Alberta Canada between April 1, 2013 and March 31, 2018 by loneliness and social isolation

	Total, n, % (95% CI)	Lonely, n, % (95% CI)	Socially isolated, n, % (95% CI)	Neither lonely or socially isolated, n, % (95% CI)
**Total**	18,191	3238 17.8 (17.2–18.4)	713 3.9 (3.6–4.2)	14,240 78.3 (77.7–78.9)
**SL Type**				
SL3	2795 15.4 (14.8–15.9)	576 20.6 (19.1–22.1)	210 7.5 (6.5–8.5)	2009 71.9 (70.2–73.6)
SL4	10,693 58.8 (58.1–59.5)	1992 18.6 (17.9–19.4)	386 3.6 (3.3–4.0)	8315 77.8 (77.0–78.6)
SL4 Dementia	4703 25.9 (25.2–26.5)	670 14.3 (13.3–15.3)	117 2.5 (2.0–2.9)	3916 83.3 (82.2–84.3)
**Female**,	11,961 65.8 (65.1–66.4)	2210 68.3 (66.7–69.9)	250 35.1 (31.6–38.6)	9501 66.7 (66.0–67.5)
**Age** (years), Mean (SD)	80.6 (12.8)	81.2 (12.5)	64.0 (14.4)	81.4 (12.1)
**Cognitive Performance Scale**				
No impairment (0)	2318 12.7 (12.3–13.2)	333 10.3 (9.2–11.3)	126 17.7 (14.9–20.5)	1859 13.1 (12.5–13.6)
Mild impairment (1–2)	10,442 57.4 (56.7–58.1)	2017 62.3 (60.6–64.0)	442 62.0 (58.4–65.6)	7983 56.1 (55.3–56.9)
Moderate impairment (3–4)	4313 23.7 (23.1–24.3)	756 23.4 (21.9–24.8)	126 17.7 (14.9–20.5)	3431 24.1 (23.4–24.8)
Severe impairment (5–6)	1118 6.2 (5.8–6.5)	132 4.1 (3.4–4.8)	19 2.7 (1.5–3.9)	967 6.8 (6.4–7.2)
**Depression Rating Scale**				
3+	3961 21.8 (21.2–22.4)	1346 41.6 (39.9–43.3)	163 22.9 (19.8–25.9)	2452 17.2 (16.6–17.8)
**ADL Self Performance Hierarchy Scale**				
Independent (0)	5903 32.5 (31.8–33.1)	1064 32.9 (31.2–34.5)	336 47.1 (43.5–50.8)	4503 31.6 (30.9–32.4)
Supervision/limited dependence (1–2)	7984 43.9 (43.2–44.6)	1412 43.6 (41.9–45.3)	311 43.6 (40.0–47.3)	6261 44.0 (43.2–44.8)
Extensive/maximal dependence (3–4)	3620 19.9 (19.3–20.5)	613 18.9 (17.6–20.3)	61 8.6 (6.5–10.6)	2946 20.7 (20.0–21.4)
Dependent/total dependence (5–6)	684 3.8 (3.5–4.0)	149 4.6 (3.9–5.3)	5 0.7 (0.1–1.3)	530 3.7 (3.4–4.0)
**IADL Difficulty Scale**				
Independent (0)	166 0.9 (0.8–1.1)	26 0.8 (0.5–1.1)	19 2.7 (1.5–3.9)	121 0.9 (0.7–1.0)
Set up/supervision (1–2)	1606 8.8 (8.4–9.2)	269 8.3 (7.4–9.3)	127 17.8 (15.0–20.6)	1210 8.5 (8.0–9.0)
Limited/extensive assistance (3–4)	2253 12.4 (11.9–12.9)	426 13.2 (12.0–14.3)	138 19.4 (16.5–22.3)	1689 11.9 (11.3–12.4)
Maximal/total dependence (5–6)	14,166 77.9 (77.3–78.5)	2517 77.7 (76.3–79.2)	429 60.2 (56.6–63.8)	11,220 78.8(78.1–79.5)
**Disease Diagnoses**				
Alzheimer’s	2634 14.5 (14.0–15.0)	416 12.9 (11.7–14.0)	40 5.6 (3.9–7.3)	2178 15.3 (14.7–15.9)
Any psychiatric diagnosis	6500 35.7 (35.0–36.4)	1324 40.9 (39.2–42.6)	492 69.0 (65.6–72.4)	4684 32.9 (32.1–33.7)
Congestive heart failure	2412 13.3 (12.8–13.8)	479 14.8 (13.6–16.0)	44 6.2 (4.4–7.9)	1889 13.3 (12.7–13.8)
Coronary artery disease	3675 20.2 (19.6–20.8)	683 21.1 (19.7–22.5)	74 10.4 (8.1–12.6)	2918 20.5 (19.8–21.2)
Dementia other than Alzheimer’s	8172 44.9 (44.2–45.7)	1470 45.4 (43.7–47.1)	182 25.5 (22.3–28.7)	6520 45.8 (45.0–46.6)
Diabetes	4139 22.8 (22.1–23.4)	763 23.6 (22.1–25.0)	174 24.4 (21.3–27.6)	3202 22.5 (21.8–23.2)
Emphysema/COPD	3793 20.9 (20.3–21.4)	752 23.2 (21.8–24.7)	169 23.7 (20.6–26.8)	2872 20.2 (19.5–20.8)
Parkinsonism	851 4.7 (4.4–5.0)	177 5.5 (4.7–6.3)	12 1.7 (0.7–2.6)	662 4.7 (4.3–5.0)
Renal failure	1524 8.4 (8.0–8.8)	280 8.7 (7.7–9.6)	35 4.9 (3.3–6.5)	1209 8.5 (8.0–9.0)
Stroke	3058 16.8 (16.3–17.4)	580 17.9 (16.6–19.2)	85 11.9 (9.5–14.3)	2393 16.8 (16.2–17.4)
**Number of conditions**				
1	127 0.7 (0.6–0.8)	14 0.4 (02–0.7)	5 0.7 (0.1–1.3)	108 0.8 (0.6–0.9)
2	1140 6.3 (5.9–6.6)	126 3.9 (3.2–4.6)	139 19.6 (16.6–22.5)	875 6.2 (5.8–6.6)
3	660 3.6 (3.4–3.9)	115 3.6 (2.9–4.2)	40 5.6 (3.9–7.3)	505 3.6 (3.3–3.9)
4	2049 11.3 (10.8–11.8)	300 9.3 (8.3–10.3)	120 16.9 (14.1–19.6)	1629 11.5 (11.0–12.0)
5+	14,086 77.7 (77.1–78.3)	2665 82.6 (81.3–83.9)	396 55.7 (52.0–59.4)	11,025 77.7 (77.0–78.4)
**Number of unique medications** (Mean, SD)	14.1 (7.2)	15.2 (7.5)	12.7 (7.2)	13.9 (7.1)
**Health instability** (CHESS score)				
No health instability (0)	10,496 57.7 (57.0–58.4)	1588 49.0 (47.3–50.8)	544 76.3 (73.2–79.4)	8364 58.7 (57.9–59.5)
Minimal/low health instability (1–2)	6969 38.3 (37.6–39.0)	1455 44.9 (43.2–46.7)	160 22.4 (19.4–25.5)	5354 37.6 (36.8–38.4)
Moderate/high health instability (3+)	726 4.0 (3.7–4.3)	195 6.0 (5.2–6.8)	9 1.3 (0.4–2.1)	522 3.7 (3.4–4.0)
**Number of falls in past 30 days** (mean, SD)	0.6 (1.4)	0.8 (1.6)	0.3 (1.1)	0.6 (1.4)
**Social Functioning**				
Not at ease interacting with others	1803 9.9 (9.5–10.4)	404 12.5 (11.3–13.6)	145 20.3 (17.4–23.3)	1254 8.8 (8.3–9.3)
Openly expresses conflict or anger with family/friends	4298 23.6 (23.0–24.2)	1007 31.1 (29.5–32.7)	184 25.8 (22.6–29.0)	3107 21.8 (21.1–22.5)
**Caregiver distress** (for those who have a caregiver)	678 3.9 (3.6–4.2)	223 7.0 (6.1–7.9)	0 (0.0)*	455 3.2 (2.9–3.5)

*These cells are empty because the operational definition of social isolation required that the resident had no primary or secondary caregiver

Overall, 65.8% (*n* = 11,961) of our cohort were female and the mean age was 80.6 (SD:12.8) years but both varied based on the presence of loneliness or social isolation. Sixty-eight percent of lonely residents (*n* = 2210) were female, compared to 35% (*n* = 250) of socially isolated residents (Table 1). The average age of lonely residents was 81 years (SD = 12.5) and for socially isolated residents was 64 years (SD = 14.4). These demographic differences between lonely and socially isolated residents persisted when we examined each individual SL level (Supplementary Tables 1, 2, 3, 4, 5, 6, 7, 8, 9 and 10).

In general, lonely residents were similar to the neither lonely nor socially isolated residents on most demographic and clinical characteristics, with the exception of being more likely to score 3+ on the DRS, have worse cognitive impairment, have 5+ chronic conditions, and higher CHESS scores. Compared to socially isolated residents, lonely residents were more cognitively impaired (CPS = 5–6: 4.1% vs. 2.7%) and more frequently scored 3+ on the DRS (41.6% vs. 22.9%). Alzheimer’s disease and other dementias were more common among lonely residents than socially isolated residents (Alzheimer’s: 12.9% vs 5.6%; other dementias: 45.4% vs. 25.5%). Sixty-nine percent of socially isolated residents (*n* = 492) had a psychiatric diagnosis compared to 41% of lonely residents (*n* = 1324). Conversely, over 80% of lonely residents had 5+ conditions (83%, *n* = 2665) compared to 56% of socially isolated (*n* = 396).

Twenty percent of socially isolated residents (*n* = 145) were not at ease interacting with others compared to 12.5% of lonely residents (Table 1). Over 30 % of lonely residents openly expressed conflict or anger with family/friends (*n* = 1007) compared to 25.8% if socially isolated residents (*n* = 184). Among those identified as lonely, the frequency of caregiver distress was double that of residents who were neither lonely nor socially isolated (7.0% vs. 3.2%). No caregiver details are available for socially isolated residents because it is a part of the operational definition.

In general, the greatest health service use was by lonely residents, followed by neither lonely nor socially isolated, and then socially isolated (Table 2). This was the case for ED visits, hospital admissions, SL level increase, and NH admissions (Supplementary Tables 1, 2, 3, 4, 5, 6, 7, 8, 9 and 10). However, for those who had an ED visit, socially isolated residents were more likely than the other resident groups to have 4+ in a year (23.2%), and if admitted to hospital, they were also the most likely to have 2+ admissions (41.2%) within year and to have the longest mean stay (mean total number of days = 29.4). At each SL level, socially isolated residents had the longer mean hospital stay in both SL3 and SL4 relative to lonely residents (Supplementary Tables 1, 2, 3, 4, 5, 6, 7, 8, 9 and 10). Compared to lonely residents, socially isolated residents were also least likely to experience ALC days in hospital (24% vs 21.6%), potentially because they were least likely to move SL levels or enter a NH. Frequency of death was similar between the lonely (8.8%) and neither group (8.6%) but approximately half as likely among the socially isolated group (4.1%); however, the socially isolated group was most likely to die in hospital compared to the others, who were more likely to die either in their SL or NH facility (Supplementary Tables 1, 2, 3, 4, 5, 6, 7, 8, 9 and 10).

**Table 2:** Health system use by SL residents in Alberta Canada between April 1, 2013 and March 31, 2018 by loneliness and social isolation

	Total *n* = 18,191	Lonely, n, % (95% CI) *n* = 3238	Socially isolated, n, % (95% CI) *n* = 713	Neither lonely or socially isolated, n, % (95% CI) *n* = 14,240
**Emergency Department Visits**				
**Any unplanned ED visits in the follow-up year**				
Yes	10,119 55.6 (54.9–56.4)	1983 61.2 (59.6–62.9)	332 46.6 (42.9–50.2)	7804 54.8 (54.0–55.6)
**If unplanned ED visit**				
1	4301 42.5 (41.5–43.5)	758 38.2 (36.1–40.4)	152 45.8 (40.4–51.1)	3391 43.5 (42.4–44.6)
2	2534 25.0 (24.2–25.9)	476 24.0 (22.1–25.9)	63 19.0 (14.8–23.2)	1995 25.6 (24.6–26.5)
3	1354 13.4 (12.7–14.0)	308 15.5 (13.9–17.1)	40 12.1 (8.6–15.6)	1006 12.9 (12.2–13.6)
4+	1930 19.1 (18.3–19.8)	441 22.2 (20.4–24.1)	77 23.2 (18.7–27.7)	1412 18.1 (17.2–19.0)
**ED visit for fall-related injury**	3742 37.0 (36.0–37.9)	752 37.9 (35.8–40.1)	107 32.2 (27.2–37.3)	2883 36.9 (35.9–38.0)
**Hospital Admissions**				
**Non-elective hospital admission in follow up year**				
Yes	6033 33.2 (32.5–33.9)	1238 38.2 (36.6–39.9)	204 28.6 (25.3–31.9)	4591 32.2 (31.5–33.0)
**If non-elective hospital admission**				
1	3858 64.0 (62.7–65.2)	781 63.1 (60.4–65.8)	120 58.8 (52.1–65.6)	2957 64.4 (63.0–65.8)
2+	2175 36.1 (34.8–37.3)	457 36.9 (34.2–39.6)	84 41.2 (34.4–47.9)	1634 35.6 (34.2–37.0)
**If non-elective hospital admission, total number of days in hospital (Mean, Median)**				
Mean (SD)	20.1 (49.8)	20.4 (56.6)	29.4 (80.3)	19.5 (45.6)
Median (IQR)	8 (4, 18)	7 (4, 17)	8 (3, 24)	8 (4, 17)
**If non-elective hospital admission, proportion who experienced time as alternate level of care status**	1438 23.8 (22.8–24.9)	297 24.0 (21.6–26.4)	44 21.6 (15.9–27.2)	1097 23.9 (22.7–25.1)

Results from the competing risk analysis are shown in Table 3. Risk of unplanned ED visit increased with loneliness (aHR = 1.10, 95% CI: 1.04–1.15) but did not increase with social isolation (aHR = 0.95, 95% CI: 0.84–1.06). An increased risk of an unplanned ED visit was also associated with a DRS score of 3+ (aHR = 1.13, 95% CI: 1.07–1.18) and a prior history of ED visits (1 visit: aHR = 1.28, 95% CI: 1.21–1.36; 2+ visits: aHR = 1.79, 95% CI: 1.71–1.88). Alzheimer Disease or other dementia were not associated with increased risk of ED visit (aHR = 0.9, 95% CI: 0.85–0.94).

**Table 3:** Fine and Gray competing risk Cox model results to test associations between loneliness and social isolation on time to first unplanned ED visit in SL residents from Alberta, Canada

	HR (95% CI)	aHR (95% CI) - Full model
**Lonely/socially isolated/neither**		
Lonely only	1.20 (1.14–1.26)	1.10 (1.04–1.15)
Socially isolated only	0.80 (0.72–0.90)	0.95 (0.84–1.06)
Neither lonely or socially isolated	1 (ref)	1 (ref)
**SL Type**		
SL3		1 (ref)
SL4		0.87 (0.82–0.92)
SL4D		0.86 (0.8–0.93)
**Female**		0.94 (0.9–0.98)
**Age, per 1 year increase**		1.01 (1.00–1.01)
**CPS Score**		
0		1 (ref)
1		0.93 (0.87–0.99)
2		0.88 (0.81–0.95)
3		0.85 (0.75–0.96)
**DRS Score**		
< 3		1 (ref)
> =3		1.13 (1.07–1.18)
**ADL Score**		
0		1 (ref)
1		1.04 (1.0–1.1)
2		0.98 (0.92–1.05)
3		0.66 (0.58–0.76)
**CHESS Score**		
0		1 (ref)
1		1.11 (1.07–1.16)
3		1.02 (0.91–1.14)
**Alzheimer’s Disease or other dementia**		0.9 (0.85–0.94)
**Any psychiatric diagnoses**		0.96 (0.92–1.01)
**ED visits in previous year**		
0		1 (ref)
1		1.28 (1.21–1.36)
2+		1.79 (1.71–1.88)
**Number of medications, per 1 increase**		1.06 (1.04–1.07)
**Number of falls, per 1 increase**		1.04 (1.02–1.05)

*HR* hazard ratio, *aHR* adjusted hazard ratio

## Discussion

In a large cohort of SL residents, we found that approximately 18% were lonely and another 4% were identified as socially isolated, and only a very small group (< 1%) were identified as both. Lonely residents were female, older, had more cognitive impairment, and a higher frequency of depression. Socially isolated residents were male, younger, and more likely to have a psychiatric disease diagnosis. The two groups (lonely and socially isolated) had little overlap in their demographic characteristics and patterns of health service use. Lonely residents had increased risk of experiencing an unplanned ED visit compared to socially isolated residents. Lonely residents had the greatest overall health service use including acute care visits and admissions and subsequent continuing care admissions.

Of all three resident groups, lonely residents had the highest frequency of health service use. For unplanned ED visits, loneliness was associated with time to visit but not social isolation, after adjusting for confounding variables. Other studies have also found that loneliness was associated with more frequent unplanned ED visits, controlling for similar variables (e.g., age, health status) [17, 18]. Loneliness has physiological effects that can accrue overtime and negatively impact physical health, mental health, and cognitive functioning [37]. Hawkley & Cacioppo reviewed the features and consequences of loneliness and suggest that the mechanisms of loneliness that contribute to negative health outcomes may include hypervigilance, inability to regulate feelings, lifestyle and health behaviours, and slowed response to stimuli [37]. All these factors have consequences for health outcomes and health service use. One study found that loneliness among community-dwelling older adults was associated with ED visits, controlling for social support and depression, suggested that the biological stress response and behavioural deficits associated with loneliness might be related to illness and subsequent ED visits [38, 39]. Studies that attempted to isolate the relationship between loneliness and health service use have noted that while it appears that loneliness is related to poorer overall health, disentangling the causal mechanisms between loneliness, health, and health service use remains a persistent challenge [40]. It is worth noting that most studies of loneliness and social isolation and ED visits have focused on community-dwelling older adults and therefore they do not consider the organizational and staffing factors in institutional settings—like SL— that add yet another layer of complexity when considering factors related to ED visits and other health service use [41]. For example, studies in institutional settings like NH have found that the decision to transfer a resident to ED is influenced by hierarchical reporting structures, perception of staff roles and expertise, and communication processes [42]. Using information available in administrative health data, we were unable to meaningfully assess how specific organizational factors in SL might influence loneliness, social isolation, and health service use. The addition of this information requires primary data collection and is an important next step in our research.

Our data highlight that loneliness and social isolation are distinct concepts [43]. Research has identified different factors that predict loneliness and social isolation, as well as different effects of loneliness and social isolation on health and health service use [44, 45]. Our findings indicate that loneliness and social isolation differ not only conceptually but also clinically. Residents who are lonely had different health outcomes and disease diagnoses compared to socially isolated residents. We also found that when socially isolated residents were admitted to hospital, they had a greater likelihood of having multiple admissions and long lengths of stay. This more intensive treatment and longer care in hospital can pose additional risks for vulnerable older adults. Screening for and developing interventions to address these forms of social disconnection (i.e., loneliness, social isolation) should consider the differences in the respective target groups and how interventions may need to differ to target specific features in each group [46]. Ensuring that care providers in the health care system are equipped with the tools to effectively identify and differentiate between structural (social isolation) and emotional (loneliness) issues is essential so that appropriate strategies for each are implemented to mitigate adverse health impacts in older adults. This in turn can contribute to the development of interventions that can help reduce unplanned ED visits.

Although SL facilities have aimed to be a ‘homelike’ alternative to NHs, there may be a gap between this goal and the reality given the prevalence of loneliness and social isolation in the setting [47, 48]. Prevalence of loneliness in our SL resident population was nearly 18% and social isolation was 4%. Data collected in Canada of older adults (65+) using the Canadian Community Health Survey (CCHS) found that approximately 20% of respondents were lonely [49]. Data from the Canadian Longitudinal Study on Aging (CLSA) found that the prevalence of loneliness was 10.2% and social isolation was 5.1%, respectively [50]. While the prevalence is relatively comparable, these national estimates are based on community-dwelling samples and exclude older adults living in congregate settings. We found that the prevalence of loneliness and social isolation decreased with each subsequent increase in SL care with the smallest prevalence found in SL4 Dementia. Based on the results of this study alone, we cannot determine if the lower prevalence in SL4 Dementia is due to issues with assessment in cognitively impaired residents, differences in the social programming available in specialized dementia units, increased caregiver engagement for residents with cognitive impairments, or some combination of all the above. One of the advantages of studying the SL sector in Alberta is the distinctions between the levels of care, making it possible to tease out the demographic and clinical differences in the resident population. Our data show differences in resident demographics and clinical characteristics, as well as loneliness and social isolation across the SL levels and these difference characteristics likely lead to (and subsequently) reinforce the experiences of either loneliness or social isolation. For example, those in SL3 were the most likely to experience social isolation but also most likely to be characterized by being younger, having a psychiatric diagnosis, and longer lengths of stay if admitted to hospital. Additional information is needed to describe the elements of care in each SL setting and how they might contribute to differences in the reporting and experience of loneliness and social isolation.

### Limitations

This study has limitations. We used a single-item measure of loneliness completed by SL facility staff rather than a validated self-report measure of loneliness. We are therefore unable to compare our findings to other studies that used more robust scales of loneliness [51]. While social isolation is not well defined or consistently operationalized, we were limited in the available variables we could select to assess residents’ available social network and support. We used residents’ first available assessment to determine loneliness and social isolation. Using only one assessment limits our ability to discern changes in loneliness and social isolation over time and if this influenced subsequent health service use patterns. Identifying differences in residents who experience chronic versus intermittent loneliness is the next stage of our work. We examined only publicly funded SL residents and did not have access to information about SL care that was paid for privately. It is unclear if there are differences in loneliness, social isolation, and health service use between public and private pay residents. This represents a limitation in our data and an area for future research. Future research should also address how the experiences of each loneliness and social isolation and their consequences differ across care settings including the community, SL, and nursing homes.

## Conclusions

In this population-based study of SL residents, we found that nearly 18% were lonely and 4% were socially isolated. Lonely residents had the greatest overall health service use and had increased risk of experiencing an unplanned ED visit compared to socially isolated residents. Our findings suggest that there are significant differences in the profiles of lonely and socially isolated residents and that contextualizing health service use by these different social characteristics may be an important tool to identify residents at differential risk of unplanned ED visits. Furthermore, we found differences across SL level which suggests that strategies to reduce unplanned ED visits must consider the contextual differences in these settings, such as staff mix, availability of activities and services, for lonely or socially isolated residents.

## Supplementary Information

**Additional file 1: Supplementary Table 1.** Continuing care system use by all SL residents in Alberta, Canada between April 1, 2013 and March 31, 2018 by loneliness and social isolation. **Supplementary Table 2.** Characteristics, functional status, and disease diagnoses of all SL3 residents in Alberta, Canada between April 1, 2013 and March 31, 2018 by loneliness and social isolation. **Supplementary Table 3.** Social functioning and support for all SL3 residents from Alberta, Canada between April 1, 2013 and March 31, 2018 by loneliness and social isolation. **Supplementary Table 4.** Health system use by all SL3 residents from Alberta, Canada between April 1, 2013 and March 31, 2018 by loneliness and social isolation. **Supplementary Table 5.** Characteristics, functional status, and disease diagnoses of all SL4 residents in Alberta, Canada between April 1, 2013 and March 31, 2018 by loneliness and social isolation. **Supplementary Table 6.** Social functioning and support for all SL4 residents in Alberta, Canada between April 1, 2013 and March 31, 2018 by loneliness and social isolation. **Supplementary Table 7.** Health system use by all SL4 residents in Alberta, Canada between April 1, 2013 and March 31, 2018 by loneliness and social isolation. **Supplementary Table 8.** Characteristics, functional status, and disease diagnoses of all SL4D residents in Alberta, Canada between April 1, 2013 and March 31, 2018 by loneliness and social isolation. **Supplementary Table 9.** Social functioning and support for all SL4D residents in Alberta, Canada between April 1, 2013 and March 31, 2018 by loneliness and social isolation. **Supplementary Table 10.** Health system use by all SL4D residents in Alberta, Canada between April 1, 2013 and March 31, 2018 by loneliness and social isolation.

## Data Availability

The datasets used and/or analyzed during the current study are currently stored in the Alberta Health Services Enterprise Data Warehouse with support provided by the Alberta SPOR Support Unit Data and Research Services team (https://absporu.ca). The data that support the findings of this study are available from the Alberta SPOR Support Unit Data and Research Services Team but restrictions apply to the availability of these data, which were used per data sharing agreement for the current study, and so are not publicly available. Data are however available from the authors (sachambe@ualberta.ca) upon reasonable request and with permission from the Alberta SPOR Support Unit Data and Research Services team (Erik.Youngson@albertahealthservices.ca).

## Abbreviations

SL: Supportive Living
NH: Nursing home
ED: Emergency department
ALC: Alternate level of care
