# Supplementary material for: Resident loneliness, social isolation and unplanned emergency department visits from supportive living facilities: a population-based study in Alberta, Canada
Source: BMC Geriatr. 2022 Jan 3;22:21. doi: 10.1186/s12877-021-02718-5 (PMC8725434; doi:10.1186/s12877-021-02718-5)
Supplement: Supplementary file 1 — Additional file 1: Supplementary Table 1. Continuing care system use by all SL residents in Alberta, Canada between April 1, 2013 and March 31, 2018 by loneliness and social isolation. Supplementary Table 2. Characteristics, functional status, and disease diagnoses of all SL3 residents in Alberta, Canada between April 1, 2013 and March 31, 2018 by loneliness and social isolation. Supplementary Table 3. Social functioning and support for all SL3 residents from Alberta, Canada between April 1, 2013 and March 31, 2018 by loneliness and social isolation. Supplementary Table 4. Health system use by all SL3 residents from Alberta, Canada between April 1, 2013 and March 31, 2018 by loneliness and social isolation. Supplementary Table 5. Characteristics, functional status, and disease diagnoses of all SL4 residents in Alberta, Canada between April 1, 2013 and March 31, 2018 by loneliness and social isolation. Supplementary Table 6. Social functioning and support for all SL4 residents in Alberta, Canada between April 1, 2013 and March 31, 2018 by loneliness and social isolation. Supplementary Table 7. Health system use by all SL4 residents in Alberta, Canada between April 1, 2013 and March 31, 2018 by loneliness and social isolation. Supplementary Table 8. Characteristics, functional status, and disease diagnoses of all SL4D residents in Alberta, Canada between April 1, 2013 and March 31, 2018 by loneliness and social isolation. Supplementary Table 9. Social functioning and support for all SL4D residents in Alberta, Canada between April 1, 2013 and March 31, 2018 by loneliness and social isolation. Supplementary Table 10. Health system use by all SL4D residents in Alberta, Canada between April 1, 2013 and March 31, 2018 by loneliness and social isolation. [file 12877_2021_2718_MOESM1_ESM.docx]

Supplementary Table 1. Continuing care system use by all SL residents in Alberta, Canada between April 1, 2013 and March 31, 2018 by loneliness and social isolation.

|  | Lonely,  n, %  (95% CI)  n=3238 | Socially isolated, n, %  (95% CI)  n=713 | Neither lonely or socially isolated,  n, %  (95% CI)  n=14240 | Total  n=18191 |
| --- | --- | --- | --- | --- |
| **Continuing Care** | | | | |
| **Admitted to higher level of SL in follow-up year** | | | | |
| Yes | 108  3.3 (2.7-4.0) | 16  2.2 (1.2-3.3) | 324  2.3 (2.0-2.5) | 448  2.5 (2.2-2.7) |
| **Admitted to nursing home in follow-up year** | | | | |
| Yes | 417  12.9 (11.7-14.0) | 35  4.9 (3.3-6.5) | 1505  10.6 (10.1-11.1) | 1957  10.8 (10.3-11.2) |
| **If admitted to nursing home, admitted from hospital** | | | | |
| Yes | 196  47.0 (42.2-51.8) | 14  40.0 (23.8-56.2) | 690  45.9 (43.3-48.4) | 900  46.0 (43.8-48.2) |
|  |  |  |  |  |
| **Death** | | | | |
| **Died during follow up year,** | | | | |
| Yes | 286  8.8 (7.9-9.8) | 29  4.1 (2.6-5.5) | 1230  8.6 (8.2-9.1) | 1545  8.5 (8.1-8.9) |
| **If died during follow up year, location of death** | | | | |
| SL facility | 120  42.0 (36.2-47.7) | 9  31.0 (14.2-47.9) | 489  39.8 (37.0-42.5) | 618  40.0 (37.6-42.4) |
| Hospital | 112  39.2 (33.5-44.8) | 18  62.1 (44.4-79.7) | 490  39.8 (37.1-42.6) | 620  40.1 (37.7-42.6) |
| Nursing home | 44  15.4 (11.2-19.6) | 2  6.9 (0.0-16.1) | 204  16.6 (14.5-18.7) | 250  16.2 (14.3-18.0) |
| Other | 10  3.5 (1.4-5.6) | 0 (0.0) | 47  3.8 (2.8-4.9) | 57  3.7 (2.8-4.6) |

Supplementary Table 2. Characteristics, functional status, and disease diagnoses of all SL3 residents in Alberta, Canada between April 1, 2013 and March 31, 2018 by loneliness and social isolation

|  | Lonely,  n, %  (95% CI) | Socially isolated,  n, %  (95% CI) | Neither lonely or socially isolated,  n, %  (95% CI) | Total,  n, %  (95% CI) |  |
| --- | --- | --- | --- | --- | --- |
| **Total** | 576  20.6  (19.1-22.1) | 210  7.5  (6.5-8.5) | 2009  71.9  (70.2-73.6) | 2795  15.4  (14.8-15.9) |  |
| **Female**, | 378  65.6 (61.8-69.5) | 64  30.5 (24.3-36.7) | 1263  62.9 (60.8-65.0) | 1705  61.0 (59.2-62.8) |  |
| **Age** (years), Mean (SD) | 78.6 (15.6) | 56.9 (13.4) | 76.5 (17.2) | 75.4 (17.4) |  |
|  |  |  |  |  |  |
| **Cognitive Performance Scale** | | | | |  |
| No impairment (0) | 84  14.6 (11.7-17.5) | 51  24.3 (18.5-30.1) | 407  20.3 (18.5-22.0) | 542  19.4 (17.9-20.9) |  |
| Mild impairment (1-2) | 382  66.3 (62.5-70.2) | 135  64.3 (57.8-70.8) | 1251  62.3 (60.2-64.4) | 1768  63.3 (61.5-65.0) |  |
| Moderate impairment (3-4) | 94  16.3 (13.3-19.3) | 22  10.5 (6.3-14.6) | 286  14.2 (12.7-15.8) | 402  14.4 (13.1-15.7) |  |
| Severe impairment (5-6) | 16  2.8 (1.4-4.1) | 0-5 | 65  3.2 (2.5-4.0) | 83  3.0 (2.3-3.6) |  |
| **Depression Rating Scale** | | | | |  |
| 3+ | 227  39.4 (35.4-43.4) | 33  15.7 (10.8-20.6) | 277  13.8 (12.3-15.3) | 537  19.2 (17.8-20.7) |  |
| **ADL Self Performance Hierarchy Scale** | | | | |  |
| Independent (0) | 247  42.9 (38.8-46.9) | 128  61.0 (54.4-67.6) | 932  46.4 (44.2-48.6) | 1307  46.8 (44.9-48.6) |  |
| Supervision/limited dependence (1-2) | 228  39.6 (35.6-43.6) | 75  35.7 (29.2-42.2) | 756  37.6 (35.5-39.8) | 1059  37.9 (36.1-39.7) |  |
| Extensive/maximal dependence (3-4) | 84  14.6 (11.7-17.5) | 7  3.3 (0.9-5.8) | 291  14.5 (13.0-16.0) | 382  13.7 (12.4-14.9) |  |
| Dependent/total dependence (5-6) | 17  3.0 (1.6-4.3) | 0-5 | 30  1.5 (1.0-2.0) | 47  1.7 (1.2-2.2) |  |
| **IADL Difficulty**  **Scale** | | | | |  |
| Independent (0) | 8  1.4 (0.4-2.3) | 12  5.7 (2.6-8.9) | 40  2.0 (1.4-2.6) | 60  2.2 (1.6-2.7) |  |
| Set up/supervision (1-2) | 68  11.8 (9.2-14.4) | 57  27.1 (21.1-33.2) | 304  15.1 (13.6-16.7) | 429  15.4 (14.0-16.7) |  |
| Limited/extensive assistance (3-4) | 86  14.9 (12.0-17.8) | 47  22.4 (16.7-28.0) | 308  15.3 (13.8-16.9) | 441  15.8 (14.4-17.1) |  |
| Maximal/total dependence (5-6) | 414  71.9 (68.2-75.6) | 94  44.8 (38.0-51.5) | 1357  67.6 (65.5-69.6) | 1865  66.7 (65.0-68.5) |  |
| **Disease Diagnoses** | | | | |  |
| Alzheimer’s | 44  7.6 (5.5-9.8) | 0-5 | 110  5.5 (4.5-6.5) | 156  5.6 (4.7-6.4) |  |
| Any psychiatric diagnosis | 235  40.8 (36.8-44.8) | 158  75.2 (69.4-81.1) | 688  34.3 (32.2-36.3) | 1081  38.7 (36.9-40.5) |  |
| Congestive heart failure | 78  13.5 (10.8-16.3) | 6  2.9 (0.6-5.1) | 276  13.7 (12.2-15.2) | 360  12.9 (11.6-14.1) |  |
| Coronary artery disease | 117  20.3 (17.0-23.6) | 17  8.1 (4.4-11.8) | 338  16.8 (15.2-18.5) | 472  16.9 (15.5-18.3) |  |
| Dementia other than Alzheimer’s | 209  36.3 (32.4-40.2) | 19  9.1 (5.2-12.9) | 663  33.0 (31.0-35.1) | 891  31.9 (30.2-33.6) |  |
| Diabetes | 125  21.7 (18.3-25.1) | 50  23.8 (18.1-29.6) | 409  20.4 (18.6-22.1) | 584  20.9 (19.4-22.4) |  |
| Emphysema/COPD | 140  24.3 (20.8-27.8) | 47  22.4 (16.7-28.0) | 392  19.5 (17.8-21.3) | 579  20.7 (19.2-22.2) |  |
| Parkinsonism | 29  5.0 (3.3-6.8) | 0-5 | 80  4.0 (3.1-4.8) | 111  4.0 (3.3-4.7) |  |
| Renal failure | 42  7.3 (5.2-9.4) | 7  3.3 (0.9-5.8) | 159  7.9 (6.7-9.1) | 208  7.4 (6.5-8.4) |  |
| Stroke | 101  17.5 (14.4-20.6) | 21  10.0 (5.9-14.1) | 359  17.9 (16.2-19.5) | 481  17.2 (15.8-18.6) |  |
| **Number of conditions** | | | | |  |
| 1 | 0-5 | 0-5 | 36  1.8 (1.2-2.4) | 42  1.5 (1.1-2.0) |  |
| 2 | 31  5.4 (3.6-7.3) | 64  30.6 (24.4-36.9) | 255  12.8 (11.3-14.2) | 350  12.6 (11.3-13.8) |  |
| 3 | 32  5.6 (3.7-7.5) | 16  7.7 (4.1-11.3) | 125  6.3 (5.2-7.3) | 173  6.2 (5.3-7.1) |  |
| 4 | 75  13.1 (10.3-15.8) | 40  19.1 (13.8-24.5) | 276  13.8 (12.3-15.3) | 391  14.1 (12.8-15.3) |  |
| 5+ | 429  74.7 (71.2-78.3) | 82  39.2 (32.6-45.9) | 1291  64.6 (62.5-66.7) | 1802  64.8 (63.0-66.5) |  |
| **Number of unique medications** (Mean, SD) | 14.2 (7.1) | 11.6 (6.3) | 12.7 (6.9) | 12.9 (6.9) |  |
| **Health instability** (CHESS score) | | | | |  |
| No health instability (0) | 250  43.4 (39.4-47.5) | 175  83.3 (78.3-88.4) | 1086  54.1 (51.9-56.2) | 1511  54.1 (52.2-55.9) |  |
| Minimal/low health instability (1-2) | 268  46.5 (42.5-50.6) | 34  16.2 (11.2-21.2) | 822  40.9 (38.8-43.1) | 1124  40.2 (38.4-42.0) |  |
| Moderate/high health instability (3+) | 58  10.1 (7.6-12.5) | 0-5 | 101  5.0 (4.1-6.0) | 160  5.7 (4.9-6.6) |  |
| **Number of falls in past** **30 days** (Mean, SD) | 0.8 (1.6) | 0.2 (0.8) | 0.6 (1.4) | 0.6 (1.4) |  |

Note: Cells with less than five cases are identified by 0-5 in all tables

Supplementary Table 3. Social functioning and support for all SL3 residents from Alberta, Canada between April 1, 2013 and March 31, 2018 by loneliness and social isolation

|  | Lonely, n, %  (95% CI)  n=576 | Socially isolated, n, %  (95% CI)  n=210 | Neither lonely or socially isolated, n, %  (95% CI)  n=2009 | Total  n=2795 |
| --- | --- | --- | --- | --- |
| **Social Functioning** | | | | |
| Not at ease interacting with others | 80  13.9 (11.1-16.7) | 35  16.7 (11.6-21.7) | 147  7.3 (6.2-8.5) | 262  9.4 (8.3-10.5) |
| Openly expresses conflict or anger with family/friends | 175  30.4 (26.6-34.1) | 44  21.0 (15.5-26.5) | 432  21.5 (19.7-23.3) | 651  23.3 (21.7-24.9) |
|  |  |  |  |  |
| **Length of time client is alone during the day^+^** | | | | |
| Long periods of time | 282  49.0 (44.9-53.0) | 65  31.0 (24.7-37.2) | 881  43.9 (41.7-46.0) | 1228  43.9 (42.1-45.8) |
|  |  |  |  |  |
| **Relationship to primary caregiver (if available)** | | | | |
| Child or child in law | 362  62.9 (58.9-66.8) | 0 (0.0)* | 1107  55.1 (52.9-57.3) | 1469  56.8 (54.9-58.7) |
| Spouse | 42  7.3 (5.2-9.4) | 0 (0.0)* | 182  9.1 (7.8-10.3) | 224  8.7 (7.6-9.8) |
| Other relative | 135  23.4 (20.0-26.9) | 0 (0.0)* | 596  29.7 (27.7-31.7) | 731  28.3 (26.5-30.0) |
| Friend/neighbor | 37  6.4 (4.4-8.4) | 0 (0.0)* | 124  6.2 (5.1-7.2) | 161  6.2 (5.3-7.2) |
|  |  |  |  |  |
| Caregiver distress (for those who have a caregiver) | 34  6.0 (4.0-7.9) | 0 (0.0)* | 51  2.6 (1.9-3.3) | 85  3.3 (2.6-4.0) |

*These cells are empty because the operational definition of social isolation required that the resident had no primary or secondary caregiver.

^+^ Other categories of this item include never or hardly ever, 1 hour, all the time. The other categories were redacted due to small cell sizes

Supplementary Table 4. Health system use by all SL3 residents from Alberta, Canada between April 1, 2013 and March 31, 2018 by loneliness and social isolation

|  | Lonely, n, %  (95% CI)  n=576 | Socially isolated, n, %  (95% CI)  n=210 | Neither lonely or socially isolated, n, %  (95% CI)  n=2009 | Total  n=2795 |
| --- | --- | --- | --- | --- |
| **Emergency Department Visits** | | | | |
| **Any unplanned ED visits in the follow-up year** | | | | |
| Yes | 375  65.1 (61.2-69.0) | 90  42.9 (36.2-49.6) | 1205  60.0 (57.8-62.1) | 1670  59.8 (57.9-61.6) |
| **If unplanned ED visit** | | | | |
| 1 | 124  33.1 (28.3-37.8) | 44  48.9 (38.6-59.2) | 379  31.5 (28.8-34.1) | 547  32.8 (30.5-35.0) |
| 2 | 81  21.6 (17.4-25.8) | 15  16.7 (9.0-24.4) | 343  28.5 (25.9-31.0) | 439  26.3 (24.2-28.4) |
| 3 | 56  14.9 (11.3-18.5) | 10  11.1 (4.6-17.6) | 157  13.0 (11.1-14.9) | 223  13.4 (11.7-15.0) |
| 4+ | 114  30.4 (25.7-35.1) | 21  23.3 (14.6-32.1) | 326  27.1 (24.6-29.6) | 461  27.6 (25.5-29.8) |
|  |  |  |  |  |
| **ED visit for fall-related injury** | 140  37.3 (32.4-42.2) | 27  30.0 (20.5-39.5) | 426  35.4 (32.7-38.1) | 593  35.5 (33.2-37.8) |
| **Hospital Admissions** | | | | |
| **Non-elective hospital admission in follow up year** | | | | |
| Yes | 229  39.8 (35.8-43.8) | 53  25.2 (19.4-31.1) | 723  36.0 (33.9-38.1) | 1005  36.0 (34.2-37.7) |
| **If non-elective hospital admission** | | | | |
| 1 | 131  57.2 (50.8-63.6) | 32  60.4 (47.2-73.6) | 439  60.7 (57.2-64.3) | 602  59.9 (56.9-62.9) |
| 2+ | 98  42.8 (36.4-49.2) | 21  39.6 (26.5-52.8) | 284  39.3 (35.7-42.8) | 403  40.1 (37.1-43.1) |
|  |  |  |  |  |
| **If non-elective hospital admission, Total number of days in hospital (Mean, Median)** | | | | |
| Mean (SD) | 20.1 (44.9) | 35.5 (124.6) | 20.0 (36.8) | 20.8 (47.4) |
| Median (IQR) | 7 (3, 16) | 5 (2, 13) | 8 (4, 18) | 7 (4, 18) |
| **If non-elective hospital admission, proportion who experienced time as alternate level of care status** | 54  23.6 (18.1-29.1) | 11  20.8 (9.8-31.7) | 180  24.9 (21.7-28.1) | 245  24.4 (21.7-27.0) |
| **Continuing Care** | | | | |
| **Admitted to higher level of SL in follow-up year** | | | | |
| Yes | 55  9.6 (7.2-12.0) | 9  4.3 (1.6-7.0) | 121  6.0 (5.0-7.1) | 185  6.6 (5.7-7.5) |
| **Admitted to nursing home in follow-up year** | | | | |
| Yes | 113  19.6 (16.4-22.9) | 0-5 | 290  14.4 (12.9-16.0) | 408  14.6 (13.3-15.9) |
| **If admitted to nursing home, admitted from hospital** | | | | |
| Yes | 46  40.7 (31.7-50.0) | 0-5 | 138  47.6 (41.8-53.3) | 185  45.3 (40.5-50.2) |
|  |  |  |  |  |
| **Death** | | | | |
| **Died during follow up year** | | | | |
| Yes | 36  6.3 (4.3-8.2) | 0-5 | 158  7.9 (6.7-9.0) | 197  7.1 (6.1-8.0) |
| **If died during follow up year, location of death** | | | | |
| SL facility | 7  19.4 (6.5-32.4) | 0-5 | 32  20.3 (14.0-26.5) | 40  20.3 (14.7-25.9) |
| Hospital | 18  50.0 (33.7-66.3) | 0-5 | 78  49.4 (41.6-57.2) | 98  49.8 (42.8-56.7) |
| Nursing home | 10  27.8 (13.2-42.4) | 0-5 | 43  27.2 (20.3-34.2) | 53  26.9 (20.7-33.1) |
| Other | 0-5 | 0-5 | 0-5 | 6  3.1 (0.7-5.5) |

Supplementary Table 5. Characteristics, functional status, and disease diagnoses of all SL4 residents in Alberta, Canada between April 1, 2013 and March 31, 2018 by loneliness and social isolation

|  | Lonely,  n, %  (95% CI) | Socially isolated,  n, %  (95% CI) | Neither lonely or socially isolated,  n, %  (95% CI) | Total,  n, %  (95% CI) |  |
| --- | --- | --- | --- | --- | --- |
| **Total** | 1992  18.6 (17.9-19.4) | 386  3.6 (3.3-4.0) | 8315  77.8 (77.0-78.6) | 10693  58.8 (58.1-59.5) |  |
| **Female**, | 1356  68.1 (66.0-70.1) | 144  37.3 (32.5-42.1) | 5609  67.5 (66.5-68.5) | 7109  66.5 (65.6-67.4) |  |
| **Age** (years), Mean (SD) | 81.7 (12.4) | 65.8 (13.4) | 82.0 (11.6) | 81.4 (12.2) |  |
|  |  |  |  |  |  |
| **Cognitive Performance Scale** | | | | |  |
| No impairment (0) | 246  12.4 (10.9-13.8) | 73  18.9 (15.0-22.8) | 1442  17.3 (16.5-18.2) | 1761  16.5 (15.8-17.2) |  |
| Mild impairment (1-2) | 1371  68.8 (66.8-70.9) | 264  68.4 (63.8-73.0) | 5501  66.2 (65.1-67.2) | 7136  66.7 (65.8-67.6) |  |
| Moderate impairment (3-4) | 324  16.3 (14.6-17.9) | 45  11.7 (8.5-14.9) | 1115  13.4 (12.7-14.1) | 1484  13.9 (13.2-14.5) |  |
| Severe impairment (5-6) | 51  2.6 (1.9-3.3) | 0-5 | 257  3.1 (2.7-3.5) | 312  2.9 (2.6-3.2) |  |
| **Depression Rating Scale** | | | | |  |
| 3+ | 749  37.6 (35.5-39.7) | 99  25.7 (21.3-30.0) | 1204  14.5 (13.7-15.2) | 2052  19.2 (18.4-19.9) |  |
| **ADL Self Performance Hierarchy Scale** | | | | |  |
| Independent (0) | 634  31.8 (29.8-33.9) | 175  45.3 (40.4-50.3) | 2947  35.4 (34.4-36.5) | 3756  35.1 (34.2-36.0) |  |
| Supervision/limited dependence (1-2) | 821  41.2 (39.1-43.4) | 172  44.6 (39.6-49.5) | 3323  40.0 (38.9-41.0) | 4316  40.4 (39.4-41.3) |  |
| Extensive/maximal dependence (3-4) | 421  21.1 (19.3-22.9) | 34  8.8 (6.0-11.6) | 1694  20.4 (19.5-21.2) | 2149  20.1 (19.3-20.9) |  |
| Dependent/total dependence (5-6) | 116  5.8 (4.8-6.9) | 0-5 | 351  4.2 (3.8-4.7) | 472  4.4 (4.0-4.8) |  |
| **IADL Difficulty**  **Scale** | | | | |  |
| Independent (0) | 16  0.8 (0.4-1.2) | 7  1.8 (0.5-3.1) | 77  0.9 (0.7-1.1) | 100  0.9 (0.8-1.1) |  |
| Set up/supervision (1-2) | 177  8.9 (7.6-10.1) | 66  17.1 (13.3-20.9) | 817  9.8 (9.2-10.5) | 1060  9.9 (9.4-10.5) |  |
| Limited/extensive assistance (3-4) | 282  14.2 (12.6-15.7) | 77  20.0 (16.0-23.9) | 1099  13.2 (12.5-14.0) | 1458  13.6 (13.0-14.3) |  |
| Maximal/total dependence (5-6) | 1517  76.2 (74.3-78.0) | 236  61.1 (56.3-66.0) | 6322  76.0 (75.1-77.0) | 8075  75.5 (74.7-76.3) |  |
| **Disease Diagnoses** | | | | |  |
| Alzheimer’s | 158  7.9 (6.8-9.1) | 15  3.9 (2.0-5.8) | 721  8.7 (8.1-9.3) | 894  8.4 (7.8-8.9) |  |
| Any psychiatric diagnosis | 838  42.1 (39.9-44.2) | 277  71.8 (67.3-76.3) | 2889  34.7 (33.7-35.8) | 4004  37.5 (36.5-38.4) |  |
| Congestive heart failure | 358  18.0 (16.3-19.7) | 32  8.3 (5.5-11.0) | 1387  16.7 (15.9-17.5) | 1777  16.6 (15.9-17.3) |  |
| Coronary artery disease | 442  22.2 (20.4-24.0) | 43  11.1 (8.0-14.3) | 1901  22.9 (22.0-23.8) | 2386  22.3 (21.5-23.1) |  |
| Dementia other than Alzheimer’s | 833  41.8 (39.7-44.0) | 93  24.1 (19.8-28.4) | 3364  40.5 (39.4-41.5) | 4290  40.1 (39.2-41.1) |  |
| Diabetes | 529  26.6 (24.6-28.5) | 101  26.2 (21.8-30.6) | 2134  25.7 (24.7-26.6) | 2764  25.9 (25.0-26.7) |  |
| Emphysema/COPD | 499  25.1 (23.2-27.0) | 98  25.4 (21.1-29.7) | 1930  23.2 (22.3-24.1) | 2527  23.6 (22.8-24.4) |  |
| Parkinsonism | 131  6.6 (5.5-7.7) | 8  2.1 (0.7-3.5) | 476  5.7 (5.2-6.2) | 615  5.8 (5.3-6.2) |  |
| Renal failure | 197  9.9 (8.6-11.2) | 21  5.4 (3.2-7.7) | 834  10.0 (9.4-10.7) | 1052  9.8 (9.3-10.4) |  |
| Stroke | 415  20.8 (19.1-22.6) | 52  13.5 (10.1-16.9) | 1606  19.3 (18.5-20.2) | 2073  19.4 (18.6-20.1) |  |
| **Number of conditions** | | | | |  |
| 1 | 3  0.2 (0.0-0.3) | 3  0.8 (0.0-1.7) | 56  0.7 (0.5-0.9) | 62  0.6 (0.4-0.7) |  |
| 2 | 59  3.0 (2.2-3.7) | 63  16.4 (12.7-20.1) | 351  4.2 (3.8-4.7) | 473  4.4 (4.1-4.8) |  |
| 3 | 58  2.9 (2.2-3.7) | 20  5.2 (3.0-7.4) | 226  2.7 (2.4-3.1) | 304  2.9 (2.5-3.2) |  |
| 4 | 155  7.8 (6.6-9.0) | 60  15.6 (12.0-19.2) | 799  9.7 (9.0-10.3) | 1014  9.5 (9.0-10.1) |  |
| 5+ | 1706  85.9 (84.4-87.4) | 235  61.0 (56.2-65.9) | 6819  82.3 (81.5-83.2) | 8760  82.2 (81.5-83.0) |  |
| **Number of unique medications** (Mean, SD) | 16.3 (7.7) | 13.5 (7.9) | 15.2 (7.3) | 15.4 (7.4) |  |
| **Health instability** (CHESS score) | | | | |  |
| No health instability (0) | 971  48.7 (46.6-50.9) | 275  71.2 (66.7-75.8) | 4791  57.6 (56.6-58.7) | 6037  56.5 (55.5-57.4) |  |
| Minimal/low health instability (1-2) | 903  45.3 (43.2-47.5) | 103  26.7 (22.3-31.1) | 3214  38.7 (37.6-39.7) | 4220  39.5 (38.5-40.4) |  |
| Moderate/high health instability (3+) | 118  5.9 (4.9-7.0) | 8  2.1 (0.7-3.5) | 310  3.7 (3.3-4.1) | 436  4.1 (3.7-4.5) |  |
| **Number of falls in past** **30 days** (Mean, SD) | 0.8 (1.6) | 0.4 (1.2) | 0.6 (1.4) | 0.6 (1.4) |  |

Supplementary Table 6. Social functioning and support for all SL4 residents in Alberta, Canada between April 1, 2013 and March 31, 2018 by loneliness and social isolation

|  | Lonely, n, %  (95% CI)  n=1992 | Socially isolated, n, %  (95% CI)  n=386 | Neither lonely or socially isolated, n, %  (95% CI)  n=8315 | Total  n=10693 |
| --- | --- | --- | --- | --- |
| **Social Functioning** | | | | |
| Not at ease interacting with others | 204  10.2 (8.9-11.6) | 87  22.5 (18.4-26.7) | 657  7.9 (7.3-8.5) | 948  8.9 (8.3-9.4) |
| Openly expresses conflict or anger with family/friends | 569  28.6 (26.6-30.6) | 106  27.5 (23.0-31.9) | 1750  21.1 (20.2-21.9) | 2425  22.7 (21.9-23.5) |
|  |  |  |  |  |
| **Length of time client is alone during the day+** | | | | |
| Long periods of time | 1192  59.8 (57.7-62.0) | 238  61.7 (56.8-66.5) | 4267  51.3 (50.2-52.4) | 5697  53.3 (52.3-54.2) |
|  |  |  |  |  |
| **Relationship to primary caregiver (if available)** | | | | |
| Child or child in law | 1393  69.9 (67.9-71.9) | 0 (0.0)* | 5686  68.4 (67.4-69.4) | 7079  68.7 (67.8-69.6) |
| Spouse | 225  11.3 (9.9-12.7) | 0 (0.0)* | 964  11.6 (10.9-12.3) | 1189  11.5 (10.9-12.2) |
| Other relative | 277  13.9 (12.4-15.4) | 0 (0.0)* | 1293  15.6 (14.8-16.3) | 1570  15.2 (14.5-15.9) |
| Friend/neighbor | 97  4.9 (3.9-5.8) | 0 (0.0)* | 372  4.5 (4.0-4.9) | 469  4.6 (4.2-5.0) |
|  |  |  |  |  |
| Caregiver distress (for those who have a caregiver) | 133  6.8 (5.7-7.9) | 0 (0.0)* | 261  3.2 (2.8-3.6) | 394  3.9 (3.5-4.2) |

*These cells are empty because the operational definition of social isolation required that the resident had no primary or secondary caregiver.

^+^ Other categories of this item include never or hardly ever, 1 hour, all the time. The other categories were redacted due to small cell sizes

Supplementary Table 7. Health system use by all SL4 residents in Alberta, Canada between April 1, 2013 and March 31, 2018 by loneliness and social isolation

|  | Lonely, n, %  (95% CI)  n=1992 | Socially isolated, n, %  (95% CI)  n=386 | Neither lonely or socially isolated, n, %  (95% CI)  n=8315 | Total  n=10693 |
| --- | --- | --- | --- | --- |
| **Emergency Department Visits** | | | | |
| **Any unplanned ED visits in the follow-up year** | | | | |
| Yes | 1250  62.8 (60.6-64.9) | 193  50.0 (45.0-55.0) | 4640  55.8 (54.7-56.9) | 6083  56.9 (56.0-57.8) |
| **If unplanned ED visit** | | | | |
| 1 | 483  38.6 (35.9-41.3) | 82  42.5 (35.5-49.5) | 1992  42.9 (41.5-44.4) | 2557  42.0 (40.8-43.3) |
| 2 | 300  24.0 (21.6-26.4) | 39  20.2 (14.5-25.9) | 1139  24.6 (23.3-25.8) | 1478  24.3 (23.2-25.4) |
| 3 | 192  15.4 (13.4-17.4) | 24  12.4 (7.8-17.1) | 661  14.3 (13.2-15.3) | 877  14.4 (13.5-15.3) |
| 4+ | 275  22.0 (19.7-24.3) | 48  24.9 (18.8-31.0) | 848  18.3 (17.2-19.4) | 1171  19.3 (18.3-20.2) |
|  |  |  |  |  |
| **ED visit for fall-related injury** | 435  34.8 (32.2-37.4) | 58  30.1 (23.6-36.5) | 1588  34.2 (32.9-35.6) | 2081  34.2 (33.0-35.4) |
| **Hospital Admissions** | | | | |
| **Non-elective hospital admission in follow up year** | | | | |
| Yes | 797  40.0 (37.9-42.2) | 124  32.1 (27.5-36.8) | 2857  34.4 (33.3-35.4) | 3778  35.3 (34.4-36.2) |
| **If non-elective hospital admission** | | | | |
| 1 | 498  62.5 (59.1-65.9) | 72  58.1 (49.4-66.8) | 1789  62.6 (60.8-64.4) | 2359  62.4 (60.9-64.0) |
| 2+ | 299  37.5 (34.2-40.9) | 52  41.9 (33.3-50.6) | 1068  37.4 (35.6-39.2) | 1419  37.6 (36.0-39.1) |
|  |  |  |  |  |
| **If non-elective hospital admission, Total number of days in hospital (Mean, Median)** | | | | |
| Mean (SD) | 18.0 (54.0) | 26.7 (59.4) | 15.9 (34.9) | 16.8 (40.9) |
| Median (IQR) | 7 (4, 17) | 8 (3, 23) | 8 (4, 16) | 8 (4, 16) |
| **If non-elective hospital admission, proportion who experienced time as alternate level of care status** | 176  22.1 (19.2-25.0) | 26  21.0 (13.8-28.1) | 590  20.7 (19.2-22.1) | 792  21.0 (19.7-22.3) |
| **Continuing Care** | | | | |
| **Admitted to higher level of SL in follow-up year** | | | | |
| Yes | 53  2.7 (2.0-3.4) | 7  1.8 (0.5-3.1) | 203  2.4 (2.1-2.8) | 263  2.5 (2.2-2.8) |
| **Admitted to nursing home in follow-up year** | | | | |
| Yes | 222  11.1 (9.8-12.5) | 23  6.0 (3.6-8.3) | 696  8.4 (7.8-9.0) | 941  8.8 (8.3-9.3) |
| **If admitted to nursing home, admitted from hospital** | | | | |
| Yes | 113  50.9 (44.3-57.5) | 8  34.8 (15.3-54.3) | 332  47.7 (44.0-51.4) | 453  48.1 (45.0-51.3) |
|  |  |  |  |  |
| **Death** | | | | |
| **Died during follow up year** | | | | |
| Yes | 199  10.0 (8.7-11.3) | 20  5.2 (3.0-7.4) | 766  9.2 (8.6-9.8) | 985  9.2 (8.7-9.8) |
| **If died during follow up year, location of death** | | | | |
| SL facility | 92  46.2 (39.3-53.2) | 0-5 | 318  41.5 (38.0-45.0) | 415  42.1 (39.1-45.2) |
| Hospital | 74  37.2 (30.5-43.9) | 13  65.0 (44.1-85.9) | 320  41.8 (38.3-45.3) | 407  41.3 (38.2-44.4) |
| Nursing home | 26  13.1 (8.4-17.8) | 0-5 | 101  13.2 (10.8-15.6) | 129  13.1 (11.0-15.2) |
| Other | 7  3.5 (1.0-6.1) | 0-5 | 27  3.5 (2.2-4.8) | 34  3.5 (2.3-4.6) |

Supplementary Table 8. Characteristics, functional status, and disease diagnoses of all SL4D residents in Alberta, Canada between April 1, 2013 and March 31, 2018 by loneliness and social isolation

|  | Lonely,  n, %  (95% CI) | Socially isolated,  n, %  (95% CI) | Neither lonely or socially isolated,  n, %  (95% CI) | Total,  n, %  (95% CI) |  |
| --- | --- | --- | --- | --- | --- |
| **Total** | 670  14.3 (13.3-15.3) | 117  2.5 (2.0-2.9) | 3916  83.3 (82.2-84.3) | 4703  25.9 (25.2-26.5) |  |
| **Female**, | 476  71.0 (67.6-74.5) | 42  35.9 (27.2-44.6) | 2629  67.1 (65.7-68.6) | 3147  66.9 (65.6-68.3) |  |
| **Age** (years), Mean (SD) | 82.0 (9.2) | 70.5 (14.3) | 82.5 (9.2) | 82.1 (9.5) |  |
|  |  |  |  |  |  |
| **Cognitive Performance Scale** | | | | |  |
| No impairment (0) | 0-5 | 0-5 | 10  0.3 (0.1-0.4) | 15  0.3 (0.2-0.5) |  |
| Mild impairment (1-2) | 264  39.4 (35.7-43.1) | 43  36.8 (28.0-45.5) | 1231  31.4 (30.0-32.9) | 1538  32.7 (31.4-34.0) |  |
| Moderate impairment (3-4) | 338  50.5 (46.7-54.2) | 59  50.4 (41.4-59.5) | 2030  51.8 (50.3-53.4) | 2427  51.6 (50.2-53.0) |  |
| Severe impairment (5-6) | 65  9.7 (7.5-11.9) | 13  11.1 (5.4-16.8) | 645  16.5 (15.3-17.6) | 723  15.4 (14.3-16.4) |  |
| **Depression Rating Scale** | | | | |  |
| 3+ | 370  55.2 (51.5-59.0) | 31  26.5 (18.5-34.5) | 971  24.8 (23.4-26.2) | 1372  29.2 (27.9-30.5) |  |
| **ADL Self Performance Hierarchy Scale** | | | | |  |
| Independent (0) | 183  27.3 (23.9-30.7) | 33  28.2 (20.1-36.4) | 624  15.9 (14.8-17.1) | 840  17.9 (16.8-19.0) |  |
| Supervision/limited dependence (1-2) | 363  54.2 (50.4-58.0) | 64  54.7 (45.7-63.7) | 2182  55.7 (54.2-57.3) | 2609  55.5 (54.1-56.9) |  |
| Extensive/maximal dependence (3-4) | 108  16.1 (13.3-18.9) | 20  17.1 (10.3-23.9) | 961  24.5 (23.2-25.9) | 1089  23.2 (22.0-24.4) |  |
| Dependent/total dependence (5-6) | 16  2.4 (1.2-3.5) | 0-5 | 149  3.8 (3.2-4.4) | 165  3.5 (3.0-4.0) |  |
| **IADL Difficulty**  **Scale** | | | | |  |
| Independent (0) | 0-5 | 0-5 | 0-5 | 6  0.1 (0.0-0.2) |  |
| Set up/supervision (1-2) | 24  3.6 (2.2-5.0) | 0-5 | 89  2.3 (1.8-2.7) | 117  2.5 (2.0-2.9) |  |
| Limited/extensive assistance (3-4) | 58  8.7 (6.5-10.8) | 14  12.0 (6.1-17.9) | 282  7.2 (6.4-8.0) | 354  7.5 (6.8-8.3) |  |
| Maximal/total dependence (5-6) | 586  87.5 (85.0-90.0) | 99  84.6 (78.1-91.2) | 3541  90.4 (89.5-91.4) | 4226  89.9 (89.0-90.7) |  |
| **Disease Diagnoses** | | | | |  |
| Alzheimer’s | 214  31.9 (28.4-35.5) | 23  19.7 (12.5-26.9) | 1347  34.4 (32.9-35.9) | 1584  33.7 (32.3-35.0) |  |
| Any psychiatric diagnosis | 251  37.5 (33.8-41.1) | 57  48.7 (39.7-57.8) | 1107  28.3 (26.9-29.7) | 1415  30.1 (28.8-31.4) |  |
| Congestive heart failure | 43  6.4 (4.6-8.3) | 6  5.1 (1.1-9.1) | 226  5.8 (5.0-6.5) | 275  5.9 (5.2-6.5) |  |
| Coronary artery disease | 124  18.5 (15.6-21.5) | 14  12.0 (6.1-17.9) | 679  17.3 (16.2-18.5) | 817  17.4 (16.3-18.5) |  |
| Dementia other than Alzheimer’s | 428  63.9 (60.2-67.5) | 70  59.8 (51.0-68.7) | 2493  63.7 (62.2-65.2) | 2991  63.6 (62.2-65.0) |  |
| Diabetes | 109  16.3 (13.5-19.1) | 23  19.7 (12.5-26.9) | 659  16.8 (15.7-18.0) | 791  16.8 (15.8-17.9) |  |
| Emphysema/COPD | 113  16.9 (14.0-19.7) | 24  20.5 (13.2-27.8) | 550  14.0 (13.0-15.1) | 687  14.6 (13.6-15.6) |  |
| Parkinsonism | 17  2.5 (1.4-3.7) | 0-5 | 106  2.7 (2.2-3.2) | 125  2.7 (2.2-3.1) |  |
| Renal failure | 41  6.1 (4.3-7.9) | 7  6.0 (1.7-10.3) | 216  5.5 (4.8-6.2) | 264  5.6 (5.0-6.3) |  |
| Stroke | 64  9.6 (7.3-11.8) | 12  10.3 (4.8-15.8) | 428  10.9 (10.0-11.9) | 504  10.7 (9.8-11.6) |  |
| **Number of conditions** | | | | |  |
| 1 | 6  0.9 (0.2-1.6) | 0-5 | 16  0.4 (0.2-0.6) | 23  0.5 (0.3-0.7) |  |
| 2 | 36  5.4 (3.7-7.1) | 12  10.3 (4.8-15.8) | 269  6.9 (6.1-7.7) | 317  6.8 (6.0-7.5) |  |
| 3 | 25  3.7 (2.3-5.2) | 0-5 | 154  3.9 (3.3-4.5) | 183  3.9 (3.3-4.5) |  |
| 4 | 70  10.5 (8.2-12.8) | 20  17.1 (10.3-23.9) | 554  14.2 (13.1-15.3) | 644  13.7 (12.7-14.7) |  |
| 5+ | 530  79.3 (76.3-82.4) | 79  67.5 (59.0-76.0) | 2915  74.5 (73.1-75.9) | 3524  75.0 (73.8-76.3) |  |
| **Number of unique medications** (Mean, SD) | 12.8 (6.5) | 12.0 (5.3) | 11.8 (5.9) | 11.9 (6.0) |  |
| **Health instability** (CHESS score) | | | | |  |
| No health instability (0) | 367  54.8 (51.0-58.5) | 94  80.3 (73.1-87.5) | 2487  63.5 (62.0-65.0) | 2948  62.7 (61.3-64.1) |  |
| Minimal/low health instability (1-2) | 284  42.4 (38.7-46.1) | 23  19.7 (12.5-26.9) | 1318  33.7 (32.2-35.1) | 1625  34.6 (33.2-35.9) |  |
| Moderate/high health instability (3+) | 19  2.8 (1.6-4.1) | 0-5 | 111  2.8 (2.3-3.4) | 130  2.8 (2.3-3.2) |  |
| **Number of falls in past** **30 days** (Mean, SD) | 0.6 (1.5) | 0.2 (0.8) | 0.6 (1.5) | 0.6 (1.5) |  |

Supplementary Table 9. Social functioning and support for all SL4D residents in Alberta, Canada between April 1, 2013 and March 31, 2018 by loneliness and social isolation

|  | Lonely, n, %  (95% CI)  n=670 | Socially isolated, n, %  (95% CI)  n=117 | Neither lonely or socially isolated, n, %  (95% CI)  n=3916 | Total  n=4703 |
| --- | --- | --- | --- | --- |
| **Social Functioning** | | | | |
| Not at ease interacting with others | 120  17.9 (15.0-20.8) | 23  19.7 (12.5-26.9) | 450  11.5 (10.5-12.5) | 593  12.6 (11.7-13.6) |
| Openly expresses conflict or anger with family/friends | 263  39.3 (35.6-43.0) | 34  29.1 (20.8-37.3) | 925  23.6 (22.3-25.0) | 1222  26.0 (24.7-27.2) |
|  |  |  |  |  |
| **Length of time client is alone during the day+** | | | | |
| Long periods of time | 241  36.0 (32.3-39.6) | 41  35.0 (26.4-43.7) | 1040  26.6 (25.2-27.9) | 1322  28.1 (26.8-29.4) |
|  |  |  |  |  |
| **Relationship to primary caregiver (if available)** | | | | |
| Child or child in law | 460  68.7 (65.1-72.2) | 0 (0.0)* | 2519  64.3 (62.8-65.8) | 2979  65.0 (63.6-66.3) |
| Spouse | 125  18.7 (15.7-21.6) | 0 (0.0)* | 840  21.5 (20.2-22.7) | 965  21.0 (19.9-22.2) |
| Other relative | 59  8.8 (6.7-11.0) | 0 (0.0)* | 440  11.2 (10.3-12.2) | 499  10.9 (10.0-11.8) |
| Friend/neighbor | 26  3.9 (2.4-5.3) | 0 (0.0)* | 117  3.0 (2.5-3.5) | 143  3.1 (2.6-3.6) |
|  |  |  |  |  |
| Caregiver distress (for those who have a caregiver) | 56  8.5 (6.4-10.6) | 0 (0.0)* | 143  3.7 (3.1-4.3) | 199  4.4 (3.8-5.0) |

*These cells are empty because the operational definition of social isolation required that the resident had no primary or secondary caregiver.

^+^ Other categories of this item include never or hardly ever, 1 hour, all the time. The other categories were redacted due to small cell sizes

Supplementary Table 10. Health system use by all SL4D residents in Alberta, Canada between April 1, 2013 and March 31, 2018 by loneliness and social isolation

|  | Lonely, n, %  (95% CI)  n=670 | Socially isolated, n, %  (95% CI)  n=117 | Neither lonely or socially isolated, n, %  (95% CI)  n=3916 | Total  n=4703 |
| --- | --- | --- | --- | --- |
| **Emergency Department Visits** | | | | |
| **Any unplanned ED visits in the follow-up year** | | | | |
| Yes | 358  53.4 (49.7-57.2) | 49  41.9 (32.9-50.8) | 1959  50.0 (48.5-51.6) | 2366  50.3 (48.9-51.7) |
| **If unplanned ED visit** | | | | |
| 1 | 151  42.2 (37.1-47.3) | 26  53.1 (39.1-67.0) | 1020  52.1 (49.9-54.3) | 1197  50.6 (48.6-52.6) |
| 2 | 95  26.5 (22.0-31.1) | 9  18.4 (7.5-29.2) | 513  26.2 (24.2-28.1) | 617  26.1 (24.3-27.9) |
| 3 | 60  16.8 (12.9-20.6) | 6  12.2 (3.1-21.4) | 188  9.6 (8.3-10.9) | 254  10.7 (9.5-12.0) |
| 4+ | 52  14.5 (10.9-18.2) | 8  16.3 (6.0-26.7) | 238  12.2 (10.7-13.6) | 298  12.6 (11.3-13.9) |
|  |  |  |  |  |
| **ED visit for fall-related injury** | 177  49.4 (44.3-54.6) | 22  44.9 (31.0-58.8) | 869  44.4 (42.2-46.6) | 1068  45.1 (43.1-47.1) |
| **Hospital Admissions** | | | | |
| **Non-elective hospital admission in follow up year** | | | | |
| Yes | 212  31.6 (28.1-35.2) | 27  23.1 (15.4-30.7) | 1011  25.8 (24.5-27.2) | 1250  26.6 (25.3-27.8) |
| **If non-elective hospital admission** | | | | |
| 1 | 152  71.7 (65.6-77.8) | 16  59.3 (40.7-77.8) | 729  72.1 (69.3-74.9) | 897  71.8 (69.3-74.3) |
| 2+ | 60  28.3 (22.2-34.4) | 11  40.7 (22.2-59.3) | 282  27.9 (25.1-30.7) | 353  28.2 (25.7-30.7) |
| **If non-elective hospital admission, Total number of days in hospital (Mean, Median)** | | | | |
| Mean (SD) | 31.0 (76.6) | 31.0 (60.2) | 30.8 (72.7) | 30.8 (73.1) |
| Median (IQR) | 9 (3, 24) | 8 (3, 30) | 9 (4, 27) | 9 (4, 26) |
| **If non-elective hospital admission, proportion who experienced time as alternate level of care status** | 67  31.6 (25.4-37.9) | 7  25.9 (9.4-42.5) | 327  32.3 (29.5-35.2) | 401  32.1 (29.5-34.7) |
| **Continuing Care** | | | | |
| **Admitted to nursing home in follow-up year** | | | | |
| Yes | 82  12.2 (9.8-14.7) | 7  6.0 (1.7-10.3) | 519  13.3 (12.2-14.3) | 608  12.9 (12.0-13.9) |
| **If admitted to nursing home, admitted from hospital** | | | | |
| Yes | 37  45.1 (34.4-55.9) | 0-5 | 220  42.4 (38.1-46.6) | 262  43.1 (39.2-47.0) |
|  |  |  |  |  |
| **Death** | | | | |
| **Died during follow up year** | | | | |
| Yes | 51  7.6 (5.6-9.6) | 6  5.1 (1.1-9.1) | 306  7.8 (7.0-8.7) | 363  7.7 (7.0-8.5) |
| **If died during follow up year, location of death** | | | | |
| SL facility | 21  41.2 (27.7-54.7) | 3  50.0 (10.0-90.0) | 139  45.4 (39.9-51.0) | 163  44.9 (39.8-50.0) |
| Hospital | 20  39.2 (25.8-52.6) | 3  50.0 (10.0-90.0) | 92  30.1 (24.9-35.2) | 115  31.7 (26.9-36.5) |
| Nursing home | 8  15.7 (5.7-25.7) | 0-5 | 60  19.6 (15.2-24.1) | 68  18.7 (14.7-22.8) |
| Other | 0-5 | 0-5 | 15  4.9 (2.5-7.3) | 17  4.7 (2.5-6.9) |
